# Twitter as a Mechanism of Knowledge Translation in Health Professions Education: An Exploratory Content Analysis

**DOI:** 10.5334/pme.1053

**Published:** 2023-12-15

**Authors:** Catherine M. Giroux, Lauren A. Maggio, Conchita Saldanha, André Bussières, Aliki Thomas

**Affiliations:** 1 Postdoctoral Fellow at Institute of Health Sciences Education at McGill University in Montreal, Quebec, and a Part-time Professor in the Faculty of Education at the University of Ottawa, Ontario, Canada; 2 Professor in the Department of Medicine at Uniformed Services University of the Health Sciences in Bethesda, Maryland, USA; 3 Research Assistant at the School of Physical and Occupational Therapy at McGill University in Montreal, Quebec, Canada; 4 Full Professor in the Department of Chiropractic at Université du Québec à Trois-Rivières and an Assistant Professor at the School of Physical and Occupational Therapy at McGill University, Quebec, Canada; 5 Associate Professor at the School of Physical and Occupational Therapy and an Associate Member of the Institute of Health Sciences Education at McGill University, Montreal, Quebec, Canada

## Abstract

**Introduction:**

Social media may facilitate knowledge sharing within health professions education (HPE), but whether and how it is used as a mechanism of knowledge translation (KT) is not understood. This exploratory study aimed to ascertain what content has been shared on Twitter using #MedEd and how it is used as a mechanism of KT.

**Methods:**

Symplur was used to identify all tweets tagged with #MedEd between March 2021 – March 2022. A directed content analysis and multiple cycles of coding were employed. 18,000 tweets were identified, of which 478 were included. Studies sharing high quality HPE information; relating to undergraduate, postgraduate, or continuing education; referring to an evidence source; and posted in English or French were included.

**Results:**

Diverse content was shared using #MedEd, including original tweets, links to peer-reviewed articles, and visual media. Tweets shared information about new educational approaches; system, clinical, or educational research outcomes; and measurement tools. #MedEd appears to be a mechanism of diffusion (n = 296 tweets) and dissemination (n = 164 tweets). It is less frequently used for knowledge exchange (n = 13 tweets) and knowledge synthesis (n = 5 tweets). No tweets demonstrated the ethically sound application of knowledge.

**Discussion:**

It is challenging to determine whether and how #MedEd is used to promote the uptake of knowledge into HPE or if it is even possible for Twitter to serve these purposes. Further studies exploring how health professions educators use the knowledge gained from Twitter to inform their educational or clinical practices are recommended.

## Introduction

Social media–platforms where users construct profiles and virtually connect with others–may facilitate knowledge sharing within health professions education (HPE) [1]. Twitter allows researchers to quickly share their findings with global audiences using searchable hashtags, images, website links, and documents; moreover, compared with other social media platforms, information shared via Twitter has been shown to improve knowledge and promote behaviour change [2,3]. The COVID-19 pandemic highlighted this use of Twitter for knowledge sharing in HPE as educators found themselves shifting from in-person clinical placements, exams, and electives to remote instruction [4,5]. Twitter created spaces to share and receive pedagogical knowledge and resources from colleagues during this transition [2,6]. For example, some educators used Twitter to share asynchronous educational modules and case studies, create social media-based journal clubs, and share ‘tweetorials’, which are tutorials on educational content within a series of 280-character tweets [6,7]. Indeed, it facilitated the creation of communities of practice, allowing health professions educators who might be new to online teaching to learn from those willing to share their resources and expertise during this period of educational disruption [4,8,9].

Despite social media’s role in sharing knowledge within HPE, little is known about its use as a mechanism of knowledge translation (KT). Knowledge translation is a concept that originated in clinical practice and health research and is increasingly applied within HPE [10]. It is typically understood as a process used to optimise the adoption, appropriate adaptation, delivery, and sustainability of effective practices and policies within specific contexts [10]. A recent scoping review [11] found that while HPE borrowed the terms ‘dissemination’ and ‘knowledge translation’ from the field of implementation science, these concepts are poorly understood and inconsistently defined. Within the HPE literature, the terms dissemination or KT are often used in passing, nested within a larger conversation on a related topic. For instance, Conde-Caballero and colleagues mention blogging’s potential for dissemination but situate the statement within the broader context of using blogs as part of a learning management system that may facilitate online teaching [12]. Similarly, Chretien and colleagues state that social media can be used for dissemination, but this brief statement is part of the larger discussion of network connectivity [13]. It is challenging to know whether social media itself is actually being used for the purposes of KT, as defined in implementation science as a more active process, or whether it is being used as a passive mechanism to diffuse knowledge. Given the growing attention on KT in HPE and the potential role of social media to facilitate this process, understanding how these platforms are used and whether they are even appropriate vehicles for KT is essential. Thus, the purpose of this exploratory study is to ascertain what content has been shared on Twitter using one specific hashtag (#MedEd) and how this content aligns with the definition and categories of knowledge translation put forth by the Canadian Institutes of Health Research (CIHR). Specifically, we sought to understand whether #MedEd was being used for the purposes of dissemination, synthesis, exchange, and the ethically sound application of knowledge.

## Conceptual framework

We adopted the CIHR definition and associated categories of KT to understand how Twitter is used in HPE. Though the CIHR definition of KT is an influential definition that was originally developed in a Canadian context, variations of this definition exist and have been adopted by global organizations [14]. CIHR describes KT as a dynamic and iterative process that includes synthesis, dissemination, exchange, and ethically sound application of knowledge to improve health, provide more effective health services and products, and strengthen the healthcare system [15]. Beyond these four categories, we also considered diffusion as a fifth category, since it is a term many use interchangeably with dissemination even though it is considered to be a more passive knowledge sharing process [16]. Table 1 presents the CIHR categories of knowledge translation and their definitions. Since diffusion is not included within the CIHR definition of knowledge translation, we have set it apart in Table 1.

**Table 1:** Canadian Institutes of Health Research Categories of Knowledge Translation Defined.


CATEGORY	DEFINITION

Knowledge Synthesis	Contextualizing and integrating research studies within the larger body of knowledge on the topic

Dissemination	Sharing research results by identifying the appropriate audience for the research findings and tailoring the message and the medium to the audience

Knowledge Exchange	Interactions between knowledge users and researchers resulting in mutual learning

Ethically Sound Application of Knowledge	The iterative process by which knowledge is actually considered, put into practice, or used to improve health and the health system. These activities must be consistent with the ethical principles and norms, social values, and legal or other regulatory frameworks.

Diffusion	Passive, unplanned, uncontrolled dissemination; primarily horizontal or mediated by peers; the potential user needs to seek out the information


CIHR clarifies that the diffusion of information requires little customization of the content to the intended audience; rather, diffusion focuses on ‘letting it happen’, that is sending the information “out to the readers/audience” without any deliberate effort at ensuring its uptake. Dissemination is a more tailored process wherein the message and vehicle is adapted to a specific audience, thereby ‘helping it happen’ [15]. Finally, ethically sound application of knowledge is yet more tailored and is aimed at moving knowledge into practice by using specific strategies that are justified in the context of the goals and target audiences, while also considering the barriers and facilitators of knowledge use. The ethically sound application of knowledge aims to ‘make it happen’ [15].

We derived a hypothesis about what may constitute social media-based dissemination and KT based on the implementation science literature and a scoping review on the topic [11]. We hypothesized that diffusion may consist of a researcher sharing a study, without tailoring the message to a particular audience. In contrast, dissemination may involve that same researcher creating a series of tweets and accompanying visual summaries (e.g., visual abstracts or infographics) to share the information from the study with a specific audience who may not have institutional access to the original article. Knowledge exchange may include such activities as a Twitter chat–using a specific hashtag–between researchers and practitioners with the goal of learning with and from each other. Knowledge synthesis, conversely, may involve crafting a Twitter thread that provides an overview of a topic and links to multiple sources of literature synthesizing the topic. Finally, the ethically sound application of knowledge may involve a researcher conducting a study, synthesizing its findings, creating a consumable message and social media campaign intended to address the facilitators and barriers to knowledge uptake, and actively working with social media-based stakeholders to put knowledge into action. Of note, these activities are not necessarily mutually exclusive (e.g., infographics may be shared as part of Twitter threads synthesizing evidence). Rather, these examples serve to illustrate the processes and levels of customization involved in each facet of knowledge translation.

## Methods

### Study design and research questions

We conducted a directed content analysis to explore whether: 1) #MedEd is being used for knowledge translation purposes on Twitter, and 2) how the content tagged with #MedEd aligns with the CIHR definition of KT. The McGill University research ethics board deemed this study exempt from further review.

### Data collection

We used Symplur, a social media tracking platform, to identify tweets tagged with #MedEd between March 2021 and March 2022. We selected this period because it was likely to yield the most recent and relevant tweets for our analysis while avoiding the predominantly pandemic-related content of 2020.

#### Inclusion criteria

We were interested in exploring all HPE content that had been tagged with #MedEd. Tweets were included if they:

- Aimed to share high quality information in HPE, which included information related to teaching strategies, learning science, pedagogy, educational research. High quality referred to resources that drew on evidence sources and not just the posters’ anecdotal experiences or opinions. This meant that they referred to a knowledge synthesis, primary research, a book, or a website as a knowledge source. Links and references were required;
- Targeted health professions educators, learners, researchers, program directors, Faculty Deans, or other decision makers (universities, ministries/departments of education);
- Related to undergraduate (didactic, clinical training); postgraduate (residency); or continuing professional development;
- Were written by healthcare professionals, educators, researchers, academics, or healthcare institutions. To be considered, the tweet author needed to provide their first and last name as well as a Twitter bio stating their position or role; and
- Were posted in English or French during the study period.

Tweets were excluded if they did not meet these criteria, or if they were posted by bots, non-academic or non-healthcare organizations (including pharma or industry), and private/anonymous accounts. No retweets were included to help manage the volume of tweets and to focus on unique content.

#### Data extraction

**Preliminary Screening**. To manage the high volume of tweets, during the study period we extracted the first 1,500 tweets per month from Symplur into Excel, for a total of 18,000 tweets. Two screeners (CG & CS) conducted a preliminary screening of all tweets for relevance, filtering tweets based on the inclusion and exclusion criteria. Both screeners independently screened two months of data to calibrate their screening approaches; they met to discuss any difficulties and resolve disagreements before dividing the remaining tweets between them. They met biweekly, after each monthly dataset (n = 1,500 tweets) had been screened, to discuss any questions or discrepancies that arose in the screening process. The first round of screening reduced the total number of tweets under consideration to 2,417.

**Secondary screening**. We conducted a second round of screening on the included tweets, verifying that each tweet aimed to share high quality information in HPE and included a link or reference to a knowledge source. Tweets were included if the information derived from peer-reviewed publications or reputable healthcare organizations. For original content (i.e., content created with the intention to be shared on Twitter, like infographics), all information needed to be appropriately referenced and contain enough detail that a viewer could easily access the original research findings. We excluded duplicate tweets. This second round of screening yielded 491 tweets for consideration.

### Data analysis

We used a directed content analysis to engage in multiple cycles of coding. Directed content analysis is used to validate or conceptually extend a theoretical framework or theory; researchers code deductively, using pre-determined categories informed by theory and the research question(s) [17]. Any tweet that did not fit the pre-determined codebook was given a new code [17]. The first cycle was deductive, using a codebook. The codebook was based on the CIHR categories of KT. It aimed to categorize tweet content as synthesis, dissemination, exchange, or ethically-sound application of knowledge. We also categorized tweets to elucidate how health professions educators are using Twitter for KT purposes, specifically noting the modalities used to share knowledge (e.g., tweetorials, news articles, videos, infographics). Subsequently, we categorized tweets based on purpose; that is, whether the tweets intended to share new educational approaches, new measurement approaches, or outcomes from research studies. The second cycle was inductive and captured in vivo codes not initially represented by our codebook. In vivo coding uses the participants’ own words or language to derive codes [18]. We also inductively identified the topic of each tweet to clarify what content was being tweeted and to what end. That is, we read each tweet and noted the topics included within each tweet (e.g., equity, diversity, inclusion; assessment; simulation, etc.), with the goal of presenting an overall picture of what knowledge was being shared using #MedEd on Twitter.

The first author (CG) met with experts in KT (AT) and information science and digital technologies (LM) and discussed the codebook and analysis strategy. All three authors piloted the codebook by independently coding 10 tweets. Following the pilot, all three researchers (CG, AT, & LM) met to compare their coding and discuss any difficulties or questions that arose. Once the codebook was finalized, the first author (CG) trained a research assistant (CS) in the coding approach; both researcher and research assistant (CG & CS) subsequently double coded the included tweets. Where disagreement occurred, they discussed the tweet, definitions, and code book to come to a final decision. If no decision could be reached, AT was consulted to provide input on the categorization. To ensure reflexivity and trustworthiness, CG and CS met throughout the coding process–every 50 Tweets–to discuss their coding, their perspectives, any areas of difficulty, and their interpretations or rival explanations [19]. The full research team was involved in peer debriefing [20]. Once all tweets had been coded, descriptive statistics were generated using IBM SPSS (v.29.0.0.0) [21].

## Results

**Figure 1:**
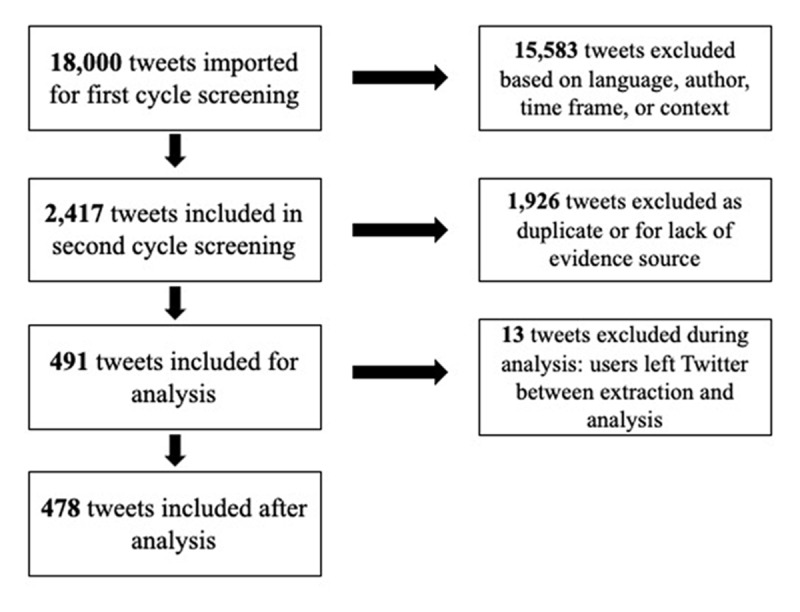
Tweets Excluded at each Stage of Screening.

We identified 18,000 tweets of which we included 478. Figure 1 depicts the number of Tweets excluded at each stage of screening.

### Overview of content tweeted using #MedEd

Diverse and multimodal content was shared using #MedEd. Specifically, the included tweets consisted of threads, a series of connected tweets by a single author on a topic; tweetorials, a series of connected tweets drafted with the intention to educate; and tweets explaining retweeted content or articles that were posted by health professions educators or researchers (n = 466); links to peer-reviewed articles (n = 290); images or other visual media (e.g., GIFs; n = 127); links to websites (e.g., the Association of American Medical Colleges website, the American Medical Association website; n = 53); links to podcasts (n = 24); conversations with or responses to colleagues (n = 17); links to videos or webinars (n = 16); infographics (n = 15); links to news articles (n = 12); links to institutional programs, initiatives, or other activities (n = 9); editable documents or resources created by individuals or organizations (e.g., through Google Drives/editable documents; n = 7); and news releases (n = 3). Seventy-three tweets consisted of quote tweets, meaning that the person sharing a tweet–originally posted by somebody else–added additional information and evidence sources as an accompanying post.

The included tweets served several purposes. They shared information about new educational approaches (n = 330). These tweets focused on new teaching or learning strategies, emerging technologies that can be used for teaching, or new ways of communicating about education. For example, one tweet read: “interested in queer curricula within #meded then look no further [sic] We are delighted to share our paper on the @thebsdj where we discuss the need to have inclusive curricula with examples ranging from communication to anatomy” [22]. Secondly, tweets shared system, clinical, or education research outcomes from research studies (n = 199). For instance, one tweet stated that “among the key implications of our work is for #meded organizations to draw attention to the concept of #structuraldistress and addressing the powerlessness that residents feel by working to enhance their agency. https://t.co/bmgaxRtxN9” [23]. Fifty-one tweets promoted awareness of measurement tools or techniques. These tweets shared information about ways to measure teaching or learning (e.g., assessment strategies, program evaluation approaches). As an example, one tweet read: “are you looking for practical guidance on asking patients for #feedback as part of a #multisource feedback program like the #MCC360? This article provides strategies that can help you feel prepared and improve feedback quality: https://t.co/WBYswc53LM. #MedEd #MedTwitter” [24].

The tweets shared during this one-year period related to numerous topics. The top three categories were teaching and learning (n = 179), social justice (n = 105), and research (n = 104). Table 2 lists the identified tweet topics. It is important to note that each tweet could contain multiple topics.

**Table 2:** Overview of Tweet Topics.


CATEGORY	EXAMPLE TOPICS	NUMBER OF TWEETS	PERCENTAGE

Teaching and Learning	Teaching strategies (e.g., experiential learning, problem-based learning, simulation, technology enhanced learning, clinical teaching, patient involvement, coaching), student assessment, feedback	179	37%

Social Justice	Equity, diversity, inclusion, antiracism, cultural competence, bias, global health	105	22%

Research	Research methods (e.g., educational, health systems) and theory (e.g., growth mindset), knowledge translation	104	22%

Wellness	Burnout, wellness, humanism, compassion	87	18%

Curriculum Design	Curriculum design, program evaluation, quality improvement, faculty development	57	12%

Learner Experience	Academic transitions, professional identity, competency based education	54	11%

Pandemic Education	COVID-19	39	8%

Knowledge, Skills, and Attitudes	Communication, leadership, clinical reasoning	18	4%

Care Delivery	Workforce, value-based care	6	1%

Unclear		4	1%


#### Level of engagement with tweeted content

Table 3 depicts the level of engagement with the included tweets, specifically the number of likes, retweets, and comments per tweet. It should be noted that the distributions are skewed, with most tweets receiving minimal (e.g., 1–9) likes, comments, or retweets. Only a few tweets received high levels of engagement (e.g., 409 likes, 115 retweets), which resulted in large variances.

**Table 3:** Level of Engagement with Included Tweets.


		NUMBER OF LIKES	NUMBER OF RETWEETS	NUMBER OF COMMENTS

Mean		9.83	3.53	0.70

Median		4.00	2.00	0.00

Mode		1.00	0.00	0.00

Std. Deviation		25.41	8.48	2.71

Minimum		0.00	0.00	0.00

Maximum		409.00	115.00	32.00

Percentiles	25	2.00	0.00	0.00

	50	4.00	2.00	0.00

	75	9.00	4.00	0.00


### Alignment with CIHR categories

Table 4 provides an overview of how the included tweets aligned with the CIHR KT categories of knowledge synthesis, dissemination, knowledge exchange, and ethically sound application of knowledge. Table 4 also indicates which tweets constitute diffusion.

**Table 4:** Overview of Tweet Alignment with CIHR Knowledge Translation Categories.


CATEGORY	NUMBER OF TWEETS	EXEMPLAR TWEETS

Diffusion	296	Fostering the Development of Master Adaptive Learners: A Conceptual Model to Guide Skill Acquisition in Medical Education https://t.co/dI5gQ73fXF #AMEE2021 #meded [25]

Knowledge Synthesis	5	[Thread] 1/ We can’t always treat. We can’t always cure. But we can always support & care with good communication. Welcome back to our #MedEd & #MedTwitter friends! Today we lay out some foundational skills of communication that you can help your learners to hone under your tutelage. https://t.co/NhulopQX3q [26]

Dissemination	164	Lockdown ruining your #MedEd? Harness the power of virtual distributed continuing professional development with Max FacDev. Podcasts + blogs + infographics @MacEmerg @WeAreCanadiEM @Kdowhos @sherbino @TchanMD @CJEMonline [27] https://t.co/7StuvvUWPZ

Knowledge Exchange	13	Dear Qualitative #meded #hpe research friends, Do you have a favourite article that is an exemplar for the use of #autoethnography in medical or health professional education research? Please share below! @LaraVarpio @LingardLorelei @ChrisWatling3 @Kori_LaDonna @ayeletkuper [28]

Ethically Sound Application of Knowledge	0	


## Discussion

This study aimed to understand if and how #MedEd is used on Twitter for KT purposes; specifically, if and how the tweets aligned with the main categories embedded within the broader process of KT, namely diffusion, knowledge synthesis, dissemination, exchange, and the ethically sound application of knowledge. Overall, our findings suggest #MedEd is used as a mechanism of diffusion, and to some extent dissemination. It remains unclear whether any tweets were part of a larger more active process of KT, focused specifically on the uptake of knowledge into practice or with the deliberate aim of bridging an identified research-to-practice gap. This may be happening but was not made explicit in the tweets. While many tweets shared only the title of an article alongside the link to that same article, others included intentionally crafted messages that were targeted to specific audiences (e.g., specific people or groups like medical students or residents), making use of visual features like emojis. This attention to how each tweet’s message was crafted may suggest that the person tweeting was attempting to increase engagement with the knowledge being shared. The included tweets leveraged several knowledge products to share information, notably peer reviewed publications and visual presentations like infographics. Many tweets included a supporting image, which is a common strategy to increase users’ engagement with the text-based tweet content [29].

With Twitter, key indicators can elucidate who is engaging with the knowledge shared and how far the message has spread. However, they largely fail to measure the impact that the knowledge has had amongst its target audience, in terms of behaviour change and/or the uptake of new knowledge into practice [30]. How users engaged with the included tweets varied widely, with most tweets receiving zero likes, retweets, or comments. Arguably, this finding suggests that social media use is removed from the principal goal of KT, which is the uptake of knowledge to bridge research-to-practice gaps. Other tweets, in contrast, received much attention. For example, a tweet on recognizing tricuspid regurgitation received as many as 409 likes and 115 retweets [31]. Of note, the people whose tweets were widely engaged with had large follower bases, suggesting they may hold an influencer status on Twitter. A social media influencer is a person who creates impact through their interactions and posts on a specific topic [32]. Whether and how social media influencers may take on some of the KT roles like knowledge brokers or local opinion leaders remains to be understood [33]. Local opinion leaders are health professionals viewed as educationally influential; opinion leadership is the degree to which an individual is able to influence others’ attitudes and behaviours in a desired way with relative frequency [34]. However, social media influencers may lack specific knowledge or expertise and therefore, their frequent postings and large follower counts may serve to proliferate the spread of misinformation online as users may mistake popularity for credibility [35,36,37].

The knowledge shared using #MedEd was topical and timely. For example, discussions of equity, diversity, and inclusion were prevalent, which is notable since the study’s data collection period followed major world events like the death of George Floyd and subsequent Black Lives Matter protests [38]. At this time, postsecondary institutions and health professions educators alike had been called upon to promote increased inclusivity for learners [39,40]. Given the estimated 17-year lag between the publication of research findings and the uptake of new knowledge into practice, platforms like Twitter may present as an opportunity for researchers to share works in progress and engage in immediately important conversations [41,42]. Future researchers could consider exploring whether or how prominent Twitter trends–like discussions related to EDI, antiracism, and implicit bias–relate to publication trends in the peer-reviewed literature.

Previous research suggests that Twitter may be an effective tool for increasing awareness of research, but not necessarily for adopting research findings in practice [41]. Importantly, it was often challenging to distinguish between diffusion and dissemination activities on Twitter throughout the analysis process. It was impossible to assess how the knowledge shared using Twitter was taken up into practice, raising questions about social media’s suitability for the active components of KT. For Twitter to be effectively leveraged as a mechanism of dissemination or even KT, health professions educators may wish to consider the following questions originally posed by Lavis and colleagues [43]: 1) What should be transferred? 2) To whom should research knowledge be transferred? 3) By whom should research knowledge be transferred? 4) How, when, and at what frequency should research knowledge be transferred? and 5) With what effect should research knowledge be transferred?

Addressing these questions may scaffold the process of crafting a message targeted to specific audiences that aligns with the goals of KT [15]. These questions may also help researchers determine who is best positioned to share knowledge and whether social media platforms, like Twitter, are even suitable vehicles to do so. When sharing knowledge, the most appropriate messenger will vary according to the target audience and message being shared [34]. Building credibility to share knowledge is time- and skill-intensive; researchers may not always be the best messenger, especially if they lack time, resources, or credibility with the target audiences [34]. Moreover, researchers are not necessarily well-equipped to create engaging or compelling content but may be able to leverage institutional communications personnel and resources [42]. It appears that a team-based approach is required for platforms like Twitter to be used as a mechanism of knowledge translation. For example, Sibley and colleagues [30] used Twitter to share knowledge with the purpose of building patient engagement capacity. To do so effectively, they created an editorial team that consisted of a KT scientist, KT and patient engagement practice leads, and knowledge brokers with 4–10 years of experience in the field. Similarly, Chambers and colleagues [42] drew on an advisory panel of 10 patient partners, alongside media experts, to create the social media campaign #ItDoesntHaveToHurt, whose purpose was to share knowledge about pediatric pain management directly with the parents and health professionals who could use it in practice. These examples from outside of HPE demonstrate the amount of work involved in using social media-based knowledge sharing to drive change. Importantly, both examples crafted a message, selected the appropriate target audience, determined who is best positioned to relay the message, and measured how the knowledge shared was taken up into practice. How social media-based knowledge sharing activities effect change in HPE itself remains to be understood.

### Strengths, Limitations, and Directions for Future Research

This study was exploratory in nature, with the aim to test whether our methodology held given our use of a directed content analysis approach to exploring Twitter posts and our use of a KT framework that may be unfamiliar to HPE. It provided an opportunity to define and categorize what KT mechanisms are at play in health professions educators’ use of social media. Moreover, social media-based research often relies on hashtag analytics (i.e., reach, impressions), platform/preference counts, and bibliometrics; we anticipate that a directed content analysis will offer new insights on Twitter’s role in knowledge sharing activities. Finally, much of the current social media research within HPE draws from lists of influencers and thus, many of the same participants. This study focuses instead on a commonly used hashtag, as well as a defined data collection period, to determine the scope of inquiry and thus had the potential to draw on a broader audience of tweeters.

Still, this study has limitations. Notably, we focused on one hashtag, which may influence the comprehensiveness of our study. We used #MedEd but were focused broadly on HPE. #MedEd is a commonly used hashtag in HPE and the exclusion of other hashtag variations or tweets posted without hashtags may exclude potentially relevant results. We focused on stand-alone tweets rather than on conversations, replies, and retweets. Therefore, additional studies that explore the interaction between users are recommended. Specifically, future research may include a network analysis to explore who is being tagged, who is interacting with whom, and the frequency of these interactions.

Additionally, we relied on the software Symplur to identify tweets tagged with #MedEd. Symplur’s algorithm influenced what tweets were made available for data extraction. Due to the volume of tweets, we chose to include only the first 1,500 tweets per month, which yielded a large number of tweets to screen, but may have excluded some relevant data. Of note, although we extracted 18,000 tweets, we were left with a much smaller number included for analysis. 15,583 tweets were excluded because they did not meet the language, authorship, or context criteria (Figure 1). Many of these tweets were posted by bots, non-academic organizations (e.g., pharma or industry), and organizations outside of health professions education using #MedEd. It is unknown if using a different approach to data collection (e.g., developing our own scraping code instead of using Symplur) would have yielded different results.

## Conclusion

#MedEd appears to be used primarily for the purposes of diffusion, and to some extent dissemination, although it is often challenging to distinguish between diffusion and dissemination activities using Twitter. Some health professions educators have used #MedEd to create Twitter threads that synthesize evidence on a topic, while others engage in knowledge exchange using the platform, but these activities appear to be less frequent than using #MedEd to support the diffusion of knowledge. Despite these uses, it is challenging to determine whether and how Twitter, and specifically #MedEd, are used to promote the uptake of knowledge into practice, or if it is even possible for them to serve these purposes at all. Further studies that explore how health professions educators use the knowledge that they gain from Twitter to inform their own educational or clinical practices are recommended.

## Disclaimers

The views expressed in this article are those of the authors and do not necessarily reflect the official policy or position of the Uniformed Services University of the Health Sciences, the Department of Defense, or the U.S. Government.
